# Agricultural Solid Wastes Based Adsorbent Materials in the Remediation of Heavy Metal Ions from Water and Wastewater by Adsorption: A Review

**DOI:** 10.3390/molecules28145575

**Published:** 2023-07-21

**Authors:** Tushar Kanti Sen

**Affiliations:** Chemical Engineering Department, College of Engineering, King Faisal University, P.O. Box 380, Al-Ahsa 31982, Saudi Arabia; tsen@kfu.edu.sa

**Keywords:** heavy metal pollution, agricultural solid waste-based adsorbents, remediation via adsorption

## Abstract

Adsorption has become the most popular and effective separation technique that is used across the water and wastewater treatment industries. However, the present research direction is focused on the development of various solid waste-based adsorbents as an alternative to costly commercial activated carbon adsorbents, which make the adsorptive separation process more effective, and on popularising the sustainable options for the remediation of pollutants. Therefore, there are a large number of reported results available on the application of raw or treated agricultural biomass-based alternatives as effective adsorbents for aqueous-phase heavy metal ion removal in batch adsorption studies. The goal of this review article was to provide a comprehensive compilation of scattered literature information and an up-to-date overview of the development of the current state of knowledge, based on various batch adsorption research papers that utilised a wide range of raw, modified, and treated agricultural solid waste biomass-based adsorbents for the adsorptive removal of aqueous-phase heavy metal ions. Metal ion pollution and its source, toxicity effects, and treatment technologies, mainly via adsorption, have been reviewed here in detail. Emphasis has been placed on the removal of heavy metal ions using a wide range of agricultural by-product-based adsorbents under various physicochemical process conditions. Information available in the literature on various important influential physicochemical process parameters, such as the metal concentration, agricultural solid waste adsorbent dose, solution pH, and solution temperature, and importantly, the adsorbent characteristics of metal ion removal, have been reviewed and critically analysed here. Finally, from the literature reviewed, future perspectives and conclusions were presented, and a few future research directions have been proposed.

## 1. Introduction

The sustainable and cost-effective remediation of water pollutants to produce clean water is a challenging task for scientists, researchers, and engineers worldwide. As per the United Nations World Water Development Report in 2020 [1], around four billion people face severe water scarcity for at least one month per year [2]. Environmental water contamination due to the large release of various potential and toxic pollutants from human activities, such as increased industrialisation, urbanisation, populations, and agricultural activities, into water bodies present a high risk to human life and aquatic environments [3]. The commonly found heavy metals ions include Cu^2+^, Ni^2+^, Zn^2+^, Cd^2+^, Pb^2+^, and Hg^2+^ ions [3,4,5]. Among them, Cd^2+^, Pb^2+^, Hg^2+^, and As^3+^ ions are the most dangerous heavy metal ions that have been identified by the World Health Organisation (WHO) [5]. Heavy metals are not biodegradable and are carcinogenic in nature. About 40% of Earth’s surface water, comprising mainly river and lake water, is being polluted by heavy metal ions primarily from the industrial and agricultural activities [6].

The major sources of heavy metal ion pollution comprise discharge from various untreated industrial effluents from refineries, coal-fired power plants, mining industries, alumina refineries, metallurgical industries, heavy chemicals, chloro-alkali industries, battery industries, dyes and pigments, fertilisers, metal smelters, paints and ceramics, tanneries, textiles, etc. [3,7]. The metal ions Cd^2+^, Pb^2+^, Hg^2+^ Cr^3+^, Cu^2+^, Mn^2+^, Fe^3+^, and Zn^2+^ are significantly toxic and pose risks to both humans and the environment [7,8,9]. There are various adverse health effects, such as diarrhoea, disorderedness, stomach problems, paralysis, various skin deceases, haemoglobinuria, vomiting, etc., that occur due to heavy metal ion contamination [3,10]. Heavy metal ions are highly toxic, hazardous to health, and non-biodegradable, and pose a high threat to the ecosystem if they are left untreated [3,11,12]. Therefore, there is an urgent need to develop ecofriendly, economically feasible technology to remove these potential pollutants from the aqueous phase [11]. Detailed information on heavy metal ion classifications, sources, and their toxicity effects have been detailed in our own previous publications [3].

**Figure 1:**
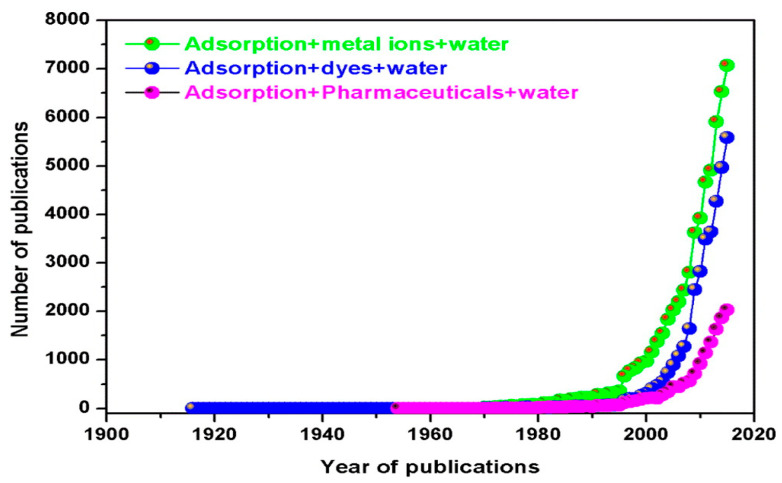
Number of adsorption publications for metal ions and organics removal. Source: taken from [3] with written permission.

A number of conventional technologies, such as chemical precipitation, oxidation, advanced oxidation, coagulation/flocculation, electrocoagulation, photo catalysis, membrane processes, reverse osmosis (RO), filtration, adsorption, solvent extraction, electroplating, ion exchange, activated sludge, and aerobic and anaerobic treatment, have been used to remove these potential pollutants from water and wastewater with varying levels of success [2,3,13,14,15,16,17,18,19,20]. All these treatment technologies have their own advantages and disadvantages. Various researchers, including the current author’s in their own reported review publication, have critically discussed the advantages and disadvantages of these different metal ion treatment technologies [7,19,21,22,23,24,25,26,27]. Among these methods, adsorption-based separation technology is one of the most effective but widely used treatment technologies for heavy metal-contaminated water and wastewater. This is due to its simple operation, design simplicity, high separation efficiency, efficiency at lower pollutant concentrations, high selectivity at the molecular level, low energy consumption, and ability to separate multiple pollutant components with minimal secondary pollution, making it a form of sustainable development [3,20,21,28]. In their previous review article, Afroze and Sen [3] reported statistical data (Figure 1) on the increasing trend of published research papers on inorganic and organic adsorption using various adsorbents since the year of 1995. The adsorptive removal of heavy metals from water and wastewater has become an essential and widely used separation technique in recent times [12].

Adsorption may be defined as the transfer of one or more solute molecules from the bulk fluid phase to the solid adsorbent surface and getting retained there. The solid that adsorbs a solute component is called the adsorbent, and the solute component that is adsorbed is termed the adsorbate. When the adsorption arises as a result of weak Van der Waals or short-range forces, it is called physical adsorption. In contrast, in the case of chemical adsorption, a chemical covalent or ionic bond formation takes place between the adsorbate and the adsorbent via electronic transfer, which is irreversible in nature [7]. There are three major steps involved at the solid/liquid interface of the adsorption process. These mechanistic steps are as follows: (a) diffusion of the solute adsorbate from the bulk aqueous phase to the surface of the adsorbent by film diffusion; (b) the adsorption at the solid/liquid interface means on the active sites of adsorbent surface; and then (c) the internal diffusion of the solute molecules within the solid adsorbent via pore diffusion or surface diffusion, or both. In simple terms, the adsorption of aqueous phase heavy metal ions involves a solid adsorbent phase and a liquid solvent phase, wherein metal ions are in the dissolved solute adsorbate molecules and are therefore part of the solid/liquid interfacial adsorption separation process. The mechanism underlying this adsorptive separation process involves chemisorption, complexation formation at the solid/liquid interface, adsorption on surface and interior pore structure of the adsorbent, ion exchange, etc., and this is due to the presence of the mass transfer concentration gradient and diffusional processes [3,14,29].

To predict the rate of adsorption and to identify the mechanisms underlying adsorption and the adsorbent’s capacity, it is vital to understand the various reported adsorption kinetic models and isotherm model equations [3,29]. In terms of the adsorption process design, the determination of various kinetic parameters is a particularly critical design parameter. Numerous kinetic models, such as the first-order and second-order reversible or irreversible kinetic models, along with the pseudo-first-order or pseudo-second-order adsorption models, have been reported and applied to batch adsorption experimental results by various researchers [2,3,30,31]. The most reported kinetic models are the pseudo-first order (PFO) and pseudo-second order (PSO) kinetic models, in which batch experimental data are fitted to these PFO and PSO models. In their critical review article, Tan and Hameed [30] mentioned that Ho [32] reviewed the applications of second-order models for adsorption systems, while Liu and Liu [33] summarised the useful kinetic models for biosorption. Surface reaction mechanism-based adsorption models have been reviewed by Plazinski et al. [34]. Alberti et al. discussed the batch and dynamic adsorption models [35]. Afroze and Sen [3] presented a compilation of reported batch adsorption results on the applicability of pseudo-second-order kinetic models for heavy metal and dye adsorption using several agricultural solid wastes, and readers are encouraged to go through this review article.

Adsorption isotherm studies are crucial for understanding the mechanisms of adsorption and for finding the maximum adsorption capacity of the adsorbent. Several adsorption isotherm models have been reported in the literature, such as the Langmuir, Freundlich, Redlich–Peterson, Tempkin, and Toth isotherm models. Of these isotherm models, the Freundlich 1906 [36] and Langmuir (1918) [37] models have been widely used in the evaluation of the adsorption process. From this research, readers are encouraged to go through the review article by Afreza and Sen [3], where the applicability of various isotherm models on the batch heavy metal adsorption process using wide ranges of agricultural solid waste-based adsorbents have been reported.

**Figure 2:**
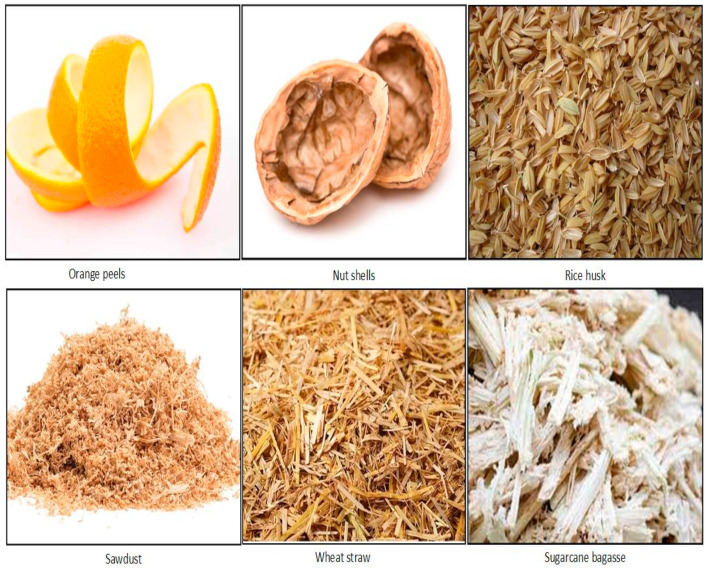
Several examples of agricultural biomass alternative adsorbents that are used in the adsorption of metal ions. Source: taken from [41] with written permission.

The adsorption process depends on the nature and the types of the adsorbent and adsorbate characteristics. The adsorbate characteristics, such as molecular weight, structure, size, charge, and solution concentration, and the adsorbent characteristics, such as particle size, surface area, surface charge, and surface functional groups are all responsible for effective adsorption [22,38]. Apart from these adsorbent-adsorbate characteristics, many physicochemical process parameters, such as the initial metal ion concentration, adsorbent dosage, contact time, solution pH, temperature, and salt concentration all significantly affect the adsorption process [3,4,27]. In the adsorptive separation process, four commonly used important adsorbents are activated carbon, zeolites or molecular sieves, natural inorganic clay minerals, silica gel, and activated alumina [39,40]. However, commercial activated carbon (CAC) is most used in the water and wastewater treatment industry due to its large porous structure, large surface area, high capacity, and the hydrophobic nature of activated carbon [3,12,38]. But coal-based CAC is costly and possesses significant regeneration issues. Therefore, the current focus of research has been shifted towards the use of various carbonaceous, lignocellulosic, and agricultural by-product solid wastes, such as fruit and vegetable wastes, leaves, seeds, tree waste, fibres, fruit peels, dates, sawdust, bark, etc., for the development of an effective adsorbent alternative to costly coal-based activated carbon. Agricultural biomasses materials, like the shells of wheat, orange peels, sunflower leaves, biochar from plant residues, activated carbon from plant residues, wood waste, bark residues, fruit wastes, and manures have been successfully used in heavy metal ion removal from water by adsorption [31]. Figure 2 shows a few agricultural by-products that are cost-effective, and function as alternative adsorbents that can be used in the adsorption of heavy metal ions.

**Figure 3:**
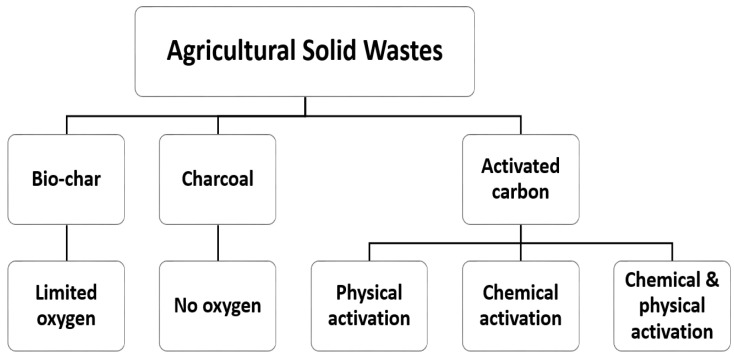
Various treatments of adsorbent materials.

**Figure 4:**
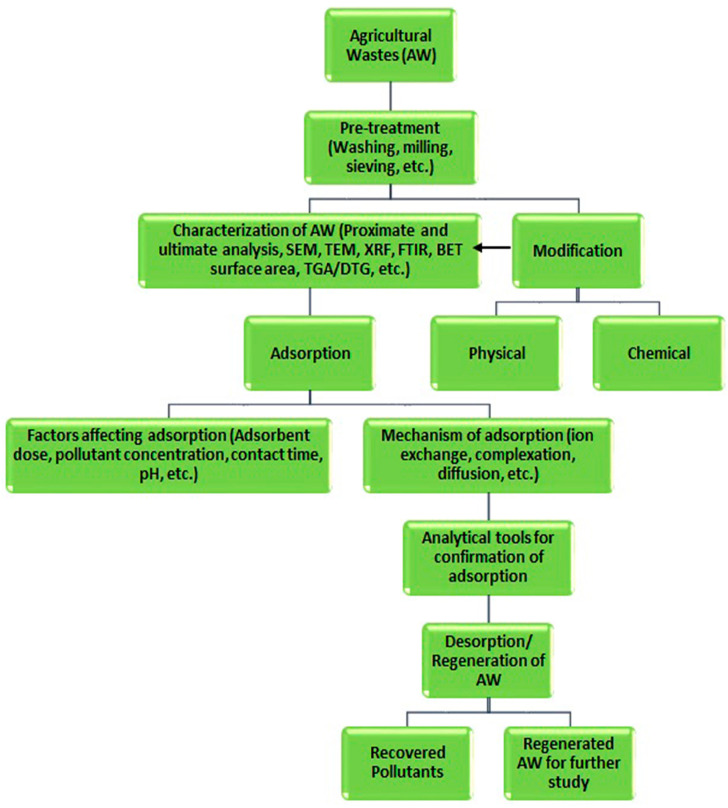
A flowchart presenting the overall adsorption process for pollutant removal from waste water. Source: taken from Ogunlalu et al. [31] with written permission.

Modified agricultural solid waste has been widely used as an effective adsorbent in the removal of various contaminations from wastewater, and this has ben attributed to their surface properties improvements. Raw biomass can be modified using acids, such as hydrochloric, phosphoric, sulfuric, nitric, citric acids etc., or alkaline solutions, such as sodium hydroxide, potassium hydroxide, zinc chloride, calcium chloride, ammonia etc., or cross-linked with other materials [3]. Chemical treatment removes natural fats, waxes, and low-molecular-weight lignin compounds from agricultural adsorbent surfaces. In recent years, the production of activated carbon, biochar, and charcoal from agricultural solid residuals is emerging as an alternative and cost-effective adsorbent with a high selectivity, porosity, and surface area, and these waste materials have naturally been available in large quantities, requires less processing time, are a renewable source, and have little or no commercial value [3,31]. Biochar is produced via the pyrolysis of biomass residues. The production and properties of these valuable adsorbents depend on the production and treatment methods, which are presented in Figure 3. Figure 4 shows a flowchart for the overall adsorption process for the removal of inorganic/organic compounds using agricultural wastes as adsorbents under various physicochemical process conditions.

In recent times, these agricultural by-products have raised environmental awareness about their safe disposal, and therefore any kind of their utilisation is considered as a win-win situation for effective solid waste management as well. Hence, this review article will provide a comprehensive compilation of all the up-to-date developments of the current state of knowledge on various batch adsorption results using a wide range of raw and modified agricultural solid waste adsorbents in the removal of heavy metal ions from water and wastewater. The significance of this review is not only the compilation and up-to-date developments of the current state of knowledge, but also the critical analysis of the recent research articles that have been published in the directions of agricultural solid waste and modified agricultural solid waste adsorbents. In this review, we have also reported and compiled the various batch heavy metal ion adsorption results under various physicochemical process parameters. Therefore, the structure of this review article began with a general introduction section comprising heavy metal ion water pollution and their sources, toxicity, and treatment methods. Emphasis has been given to agricultural by-product-based adsorbents for the removal of aqueous phase heavy metal ions through adsorption under various process conditions. Finally, the knowledge gap between the future perspectives and the future directions have been presented.

## 2. Characteristics of the Role of Adsorbents and Agricultural Waste-Based Adsorbents in Heavy Metal Adsorption

The current research has primarily been driven towards using lignocelluloses, and carbonaceous, agricultural, and forest-based adsorbents for water decontamination, including metal decontamination using an adsorption alternative to the costly CAC. These materials are available locally in large quantities and are almost priceless, with a minimum pre-treatment cost for improvements in terms of their effectiveness, efficiency, and environmental friendliness, and are an alternative adsorbent to the costly CAC. Further, agricultural solid waste adsorbent materials require minimal pre-treatment operations, such as washing, drying, grinding, or minor chemical treatments [42]. The adsorption capacity of an adsorbent plays a vital role in the selection of effective adsorbents in the removal of aqueous phase pollutants, which is either determined experimentally or theoretically using various isotherms and kinetic models. The metal adsorption at the solid/liquid interface is highly dependent on many physicochemical process parameters, such as metal ion concentration, solution pH, temperature, adsorbent dose etc., and hence the adsorption capacity was discussed and reviewed in the next section. For example, Gumus et al. [43] reported that the leaf biomass of *Laurus nobilis* is an effective adsorbent in the removal of Cd^2+^, Cu^2+^, Pb^2+^, and Zn^2+^ toxic metal ions from its aqueous solution and strong functions of temperature and solution pH with the adsorption capacity as the pH increases. A theoretical maximum Cr^6+^ adsorption capacity of 70.49 mg/g for data palm empty fruit bunch biomass was obtained at an optimum solution pH of 2 and a temperature of 30 °C [44]. Rice bran and rice straw adsorbents were successfully used to remove aqueous phase Cu^2+^ metal ions and their reported maximum adsorbent capacities were found to be 21 mg/g and 18.4 mg/g, respectively [45]. Similarly, the metal ions Pb^2+^ and Cr^6+^ were also effectively removed from water using the peanut shell residue adsorbent [46,47]. The same peanut shell residue biomass was effectively used to remove the aqueous phase from the Cr^3+^, Cu^2+^, and Pb^2+^ ions with an adsorption capacity of 7.7 mg/g, 10.2 mg/g, and 29.1 mg/g, respectively [48]. Afroze et al. [49] successfully developed a eucalyptus bark-based adsorbent for the removal of heavy metal ions from water. Ahmed and Danish [50] reviewed the raw and treated avocado waste-based effective adsorbents used in heavy metal ion removal under various conditions. Anastopoulos et al. [51] reviewed and compiled various coffee adsorbents, such as coffee grounds, coffee residues, spent coffee grains, and coffee husks in the removal of aqueous phase heavy metal ions under various experimental conditions. Hence, while a large number of reported metal adsorption results through various raw or treated/modified agricultural solid waste-based processes have been deemed as effective, cost-effective alternative adsorbents include fruit wastes, such as lemon peel [52], durian peel [53], banana peel, Kuwai peel [54], raw pomegranate peel [55], watermelon shell [56], and coconut coir [57], along with various tree leaves, such as *Artocarpus odoratissimus* leaves [58], and *Colocation esculenta* leaves [59]. All these articles have also reported on the effects of various factors on heavy metal ion adsorption kinetics and equilibrium adsorption by agricultural wastes and their maximum adsorption capacity. Raw and chemically activated various agricultural wastes, such as jackfruit, rice husk, pecan shell, bamboo, pine leaves, pinecone, eucalyptus bark, hazelnut shell, maize cob or husk, castor hull etc., are also reported effective adsorbents in the removal of aqueous phase heavy metal ions [3,31]. There are a couple of reported review articles available in the literature, such as those by Ahmed and Danesh, [50]; Saukat et al. [42]; Ogunlalu et al. [31]; Afroze and Sen; [3]; and Sulyman et al. [60] on aqueous phase heavy metal ion removal through selective agricultural solid waste-derived adsorbents. Their maximum adsorbent capacities have been reported, and readers are encouraged to go through these articles. Table 1 presents the compilation of various reported results on the maximum adsorption capacity of various agricultural by-products in the removal of heavy metals from water during the last 10-year period of 2012–2022.

The effectiveness and adsorbent capacity depend on the adsorbent’s size, shape, and morphological and chemical structure, including surface characteristics such as the surface area, pore volume, point of zero charge (pH_pzc_), bulk density, and the presence of surface functional groups [49,97]. The presence of surface functional groups in agricultural by-product adsorbent surfaces, such as carbonyl, phenolic, acetamido, alcoholic, amino groups etc., undergo strong interactions with heavy metal ions under physicochemical process conditions to form metal complexes or chelates. Adsorption is a reaction, and the rate of adsorption increases with the adsorbent surface area, shape, and surface charge, respectively. Table 2 represents the effects of various adsorbent characteristic parameters on heavy metal ion adsorption from some of the more recently published research articles [3].

## 3. Batch Metal Ion Adsorption by Agricultural Solid Waste Biomass Adsorbents under Various Physicochemical Process Parameters

In this section, the effects of the important process parameters, such as metal ion concentration, contact time, adsorbent load, pH, and temperature on the adsorbent capacity towards metal ion adsorption has been reviewed and discussed below. The identification and optimisation of these process parameters were generally determined through batch adsorption studies prior to pilot-scale continuous adsorption operation.

### 3.1. The Effects of the Initial Metal Ion Concentration and the Contact Time

**Figure 5:**
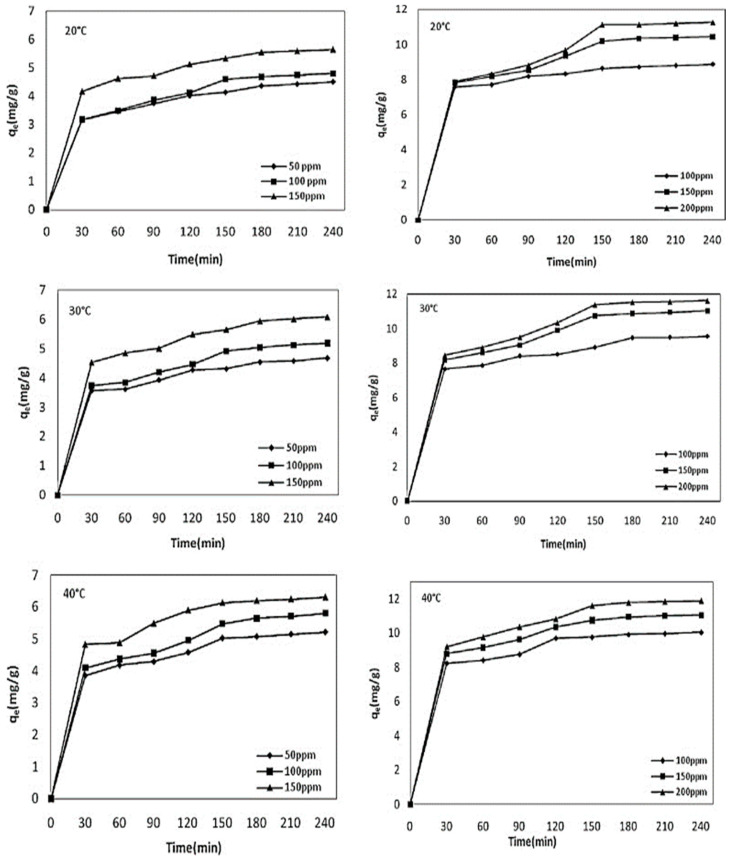
The effects of the contact time and initial metal ion concentrations of Ni^2+^ and Co^2+^ on the amount of adsorption by the almond shell biochar adsorbent at temperatures of 20, 30, and 40 °C, respectively. Source: taken from [112] with written permission.

To understand the adsorbate load and their optimum load concentration, a wide range of initial adsorbate metal ion concentrations has been examined across various reported batch adsorption studies [3,104]. Generally, with the increase in the initial adsorbate heavy metal ion concentration, the percentage removal efficiency of the carbon-based adsorbents initially increased up to a certain level and then decreased [20,105,106]. A higher solute concentration increases the competition due to the presence of excess solutes in the system to adhere with an adsorbent surface, which subsequently reduces the overall removal efficiency of the system [4,27,49,100,107]. The adsorbate or solute offers the driving force in terms of the concentration gradient to overcome the mass transfer resistance. Increasing the initial adsorbate concentration leads to the decrease in the percentage of adsorbate metal removal and an increase in the amount of heavy metal ions adsorbed per gram of adsorbent (*q_t_*). At lower concentration ranges, the available adsorbent sites are occupied by adsorbate molecules and hence increase the adsorption capacity [49]. Sometimes the adsorption process slows down due to the steric repulsion between the solute molecules [108]. Generally, the higher percentage of heavy metal removal decreases with the metal ion concentration; in this research direction, readers are encouraged to go through these various recently reported review articles [3,11,20,69,104]. The percentage removal of Zn^2+^ metal ions by the sorghum hull adsorbent was found to have decreased from 50.98% to 12.8% for the metal ion concentration range of 10–50 mg/L, respectively [86]. With the increase in the initial metal ion concentration from 25 to 150 mg/L, the percentage of adsorption of the rice husk decreased from 90.8% to 60.85% for Cr^2+^, 96.12% to 65.42% for Pb^2+^, and from 94.36% to 66.83% for Zn^2+^, respectively [20,109]. Similarly, it was reported by Ding et al. [110] that the maximum hickory wood biochar adsorbent capacity for the Cd^2+^, Zn^2+^, Ni^2+^, and Cu^2+^ metal ions was increased with the metal ion concentrations of 2–100 mg/L, respectively [7]. Yargic et al. [111] reported on the batch Cu^2+^ adsorption studies by the chemically-treated tomato waste where the percentage of metal ion removal decreased with the increase in the initial metal ion concentration, and the adsorbed amount of metal (*q_e_*) per gram of adsorbent increased with the initial metal ion concentration. Similarly, Kilic et al. [112] presented the variation between the adsorptive capacities of Ni^2+^ and Co^2+^, *q_e_* (mg/g), by almond shell biochar with the Ni^2+^ and Co^2+^ metal ion concentration ranges of 50–150 ppm and 100–200 ppm, respectively, under various temperatures, which are presented in Figure 5. As shown in Figure 5, the metal ion adsorption increased with time and followed the three step process with an initial fast reaction rate period followed by a slow rate, ending with the attainment of an equilibrium stage at 240 min [112]. A further amount of metal ion adsorption (*q_e_* (mg/g)) was increased with the increased temperature, which is also shown in Figure 5. The adsorption capacity of the Hass avocado shell (HAS) adsorbent for Ni^2+^ increased from 5.63 to 107.26 mg per gram, respectively, with the increase in the metal ion concentration [50].

Generally, the percentage removal of aqueous phase pollutants by initial adsorption increases with the contact time, and then slowly reaches a steady-state saturation level. It may present in the form of either a two-stage or multistage adsorption process [3,4,14,49,63]. Therefore, adsorption kinetic studies are important for obtaining crucial knowledge on the speed of the reaction and the equilibrium time for maximum adsorption achievement, as well as to know the kinetic parameters required for the adsorber design.

### 3.2. Effects of the Adsorbent Dose

**Figure 6:**
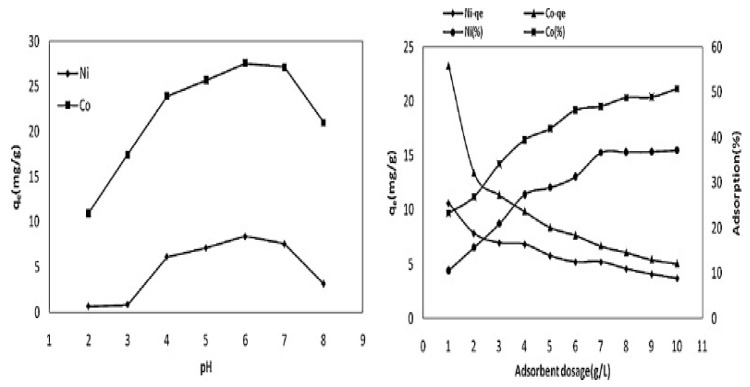
Effects of the solution pH and adsorbent dosages on Ni^2+^ and Co^2+^ adsorption. Source: taken from Kilic et al. [112] with written permission.

For the successful design, development, and scale-up of a continuous adsorption column, the knowledge of the adsorption capacity of the adsorbent is essential. The effect of an adsorbent dose on heavy metal adsorption in a solution indicates its adsorption capacity, which also depends on the available active sites on the adsorbent’s surface for adsorption [63,97]. In general, the adsorption capacity *q_e_* (mg/g) decreases with the increase in the adsorbent dose, whereas the percentage removal of metal ions increases along with the increase in the adsorbent dose [97,113]. A high adsorption capacity indicates that the adsorption process is running with a lower adsorbent dose/load. At higher adsorbent doses, there are maximum available active sites for adsorption and hence higher percentage removals of the adsorptive metal ions takes place at higher adsorbent dosages [3]. However, with a lower adsorption capacity, the removal percentage of pollutants increases rapidly and then slows down as the dose is reduced [50,114]. Much of the information presented in the literature supports these findings, such as Kılıç et al. [112], who reported from their batch adsorption study that the percentage adsorptive removal of the Ni^2+^ and Co^2+^ metal ions by the almond shell biochar increased from 10% to 38%, and from 25% to 50%, with the increase in the adsorbent doses from 1 to 10 g/L, respectively. In contrast, Ni^2+^ and Co^2+^ adsorbent’s capacities, *q_e_* (mg/g), were decreased from 10 mg/g to 3 mg/g, and from 24 mg/g to 7 mg/g, respectively, for which their results are presented in Figure 6.

Afroze et al. [49] also reported similar results for Zn^2+^ adsorption by modified eucalyptus *sheathiana* bark biomass, and it was found that their adsorbent capacity, *q_e_* (mg/g), decreased from 72.52 mg g^−1^ to 17.57 mg g^−1^ with the increase in the adsorbent doses from 0.01 g to 0.03 g, respectively [4]. There are also a few reported results on the same trends, i.e., with increases in the adsorbent dose accompanied with a decrease in the percentage of metal adsorption [115]. Imran-Shaukat et al. [42] reviewed and presented a compilation list on the variation of the adsorptive capacities of various amounts/loads of different agricultural biomass groups (such as bark, husk, leaves, peels, seeds, and straw) towards heavy metal ion (including Cd^2+^, Co^2+^, Cr^2+^, Cu^2+^, Mn^2+^, Ni^2+^, Pb^2+^, and Zn^2+^) adsorption, and critically analysed their comparative results at high, medium, and low adsorbent doses. When the amount of adsorbent mass in a fixed-volume solution is below the optimum value, the removal of metal ions is also low due to the lower number of available active sites for adsorption [69]. Table 3 presents an updated compilation of the selected reported results on the effect of adsorbent dosage in the removal of aqueous phase heavy metals using agricultural waste biomass during the last 10-year period [3].

### 3.3. Influential Effect of the Solution pH

The variation of solution pH plays a major role in changing the adsorbent surface charges, degree of ionisation, and metal speciation in solution, and hence causes changes to the adsorption capacity during the adsorption process [98,123]. Therefore, changes in the solution pH facilitate the adsorbent site dissociation and adsorbate solution chemistry, such as hydrolysis, surface complex formation, redox reactions, and precipitation, which are all strongly influenced by the pH [124]. The protonation and deprotonation of both functional groups in the adsorbent and adsorbate compound will produce different surface charges/zeta potential in the solution depending on the system’s pH [125]. Adsorbent capacity depends on its point of zero charge (pH_pzc_), and hence the surface charge. The point of zero charge (pzc) or the isoelectric point (iep) is defined as a particular pH where the surface charge becomes zero, i.e., where the extent of the adsorption of the positively charged species equals that of the negatively charged species. The point of zero charge (pH_pz_) of various raw, treated, or modified agricultural biomass-based adsorbents was determined by many investigators to obtain a better understanding of the adsorptive removal mechanism [49,126,127,128]. Generally, at lower acidic solutions, where pH < pH_pzc_, the adsorbent surface becomes positively charged and hence less metal cation adsorption takes place due to electrostatic repulsion between the positive cations and the positive surface-binding sites. Whereas, at pH > pH_pzc_, the surface becomes negatively charged and favours metal cation adsorption. However, at a higher basic pH, metal complex formation occurs resulting in precipitative separation instead of adsorptive metal ion separation [60]. For example, at a solution pH < 6.0, Pb (NO_3_)_2_ in solution predominately exists as Pb^2+^ ions. Meanwhile, with an increasing solution pH, for example at pH = 8, Pb (OH)^+^ formation occurs, and at pH = 11, it will precipitate as Pb (OH)_2_ [49,129]. Therefore, cationic species adsorption is favoured at pH > pH_pzc_ due to the presence of the functional groups, such as the OH^−^, and COO^−^ groups, while anionic adsorbate adsorption is favoured at pH < pH_pzc_ due to the presence of H^+^ ions [113,130]. An electrical double layer at the solid/liquid interface is formed by the adsorbing counter ions from the aqueous solution to its adsorbent surface. Overall, the adsorbent surface functional groups/surface charges and the chemical nature of adsorbates at a solution pH strongly influence the adsorption behaviour and capacity. In their review article, Ahmad and Danish [50] mentioned that Mallampati et al. [72,131] reported the results of the solution pH effect on the adsorptive removal of the aqueous phase Pb^2+^, Ni^2+^, and Cr_2_O_7_^2−^ ions with the avocado peel adsorbent. They found that the percentage removal of the cationic Pb^2+^ and Ni^2+^ adsorption was increased with the increase in the solution pH, whereas the adsorption of anionic Cr_2_O_7_^2−^ was decreased with the same increasing solution pH. Abbar et al. [79] presented the batch adsorption experimental results on the effects of the solution pH on Cu^2+^, Ni^2+^, and Zn^2+^ adsorption by the flax fibre tows (FFTs) adsorbent in the solution pH range from 1.6 to 8.5, respectively, for all metal ions. To investigate the effects of the solution pH on metal ion precipitation, Abbar et al. [79] presented the experimental results without adsorbent. It was found that the percentage removal of all three metal cations increased with the solution pH and attained a maximum value at an optimum pH, and thereafter decreased with the further increase in the solution pH. The maximum percentage removal of the Pb^2+^ and Cu^2+^ ions occurred in the solution pH range of 4–6, whereas for Zn^2+^ metal ions, the maximum values were observed at the solution pH of 7, respectively. At a higher pH, lead, copper, and zinc metal ions precipitate as hydroxides and reduce the rate of adsorption and hence reduce their removal capacity as well [79]. Similarly, Kilic et al. [112] reported that the amount of Ni^2+^ and Co^2+^ adsorption, *q_e_* (mg/g), by the almond shell biochar was increased from solution pH 2 to 6, and then decreased with the increasing solution pH, as shown in Figure 6. The deprotonation of the agricultural solid waste-based adsorbent typically takes place at a solution pH higher than pH_zpc_, and the surface becomes more negatively charged due to the presence of the stretching hydroxyl (–OH) and carboxyl (–COOH) functional groups [4]. Therefore, more adsorption of the cationic metal ions takes place mainly through the electrostatic force of attraction mechanism. Dawood and Sen [4], reported a similar trend in Ni^2+^ adsorption using the pinecone biochar adsorbent. At a low solution, the pH tends to decrease the adsorption capacity of the cations onto the adsorbent due to the presence of hydronium (H_3_O^+^) ions competing with the cationic metal ions for the available adsorption sites [7], accompanied with the fact that similar charges repel each other [50]. However, a lower, acidic solution pH favours anionic ion adsorption more, and this is because of the positively charged adsorbent surface and the opposite counter anions adsorption mechanism.

### 3.4. Effects of the Temperature and Thermodynamics of Adsorption

Temperature plays an important role in the adsorption of metal ions associated with the thermodynamics of the adsorption process. Temperature was found to be another significant physiochemical process parameter that influences the adsorption/biosorption mechanism and hence the equilibrium adsorbent capacity [3,42,132]. Different metal ions and different adsorbents have different responses to the system’s temperature [7,42,133]. Temperature induces various changes in the thermodynamic parameters, such as changes in the Gibb’s free energy (Δ*G*^0^), enthalpy (Δ*H*^0^), and entropy (Δ*S*^0^), for the heavy metal ion adsorption by the agricultural solid waste-based adsorbents, which can be determined by the following two equations [134]:

$$\Delta G^{0}=\Delta H^{0}-T\Delta S^{0}$$

and

$$log\left(1000\frac{q_{e}}{C_{e}}\right)=\frac{\Delta S^{0}}{2.303R}+\frac{-\Delta H^{0}}{2.303RT}$$

where *q_e_* is the amount of metal ion adsorbed per unit mass adsorbent (mg/g), *C_e_* is the equilibrium concentration (mg/L), *T* is the temperature in *K*, and *R* is the universal gas constant (8.314 J/molK).

Shaukat et al. [42] recently reviewed and reported the temperature effects on the agricultural waste biomass adsorption efficiency for various heavy metal ions under three temperature levels: high: 45 °C < x ≤ 60 °C, medium: 30 °C < x ≤ 45 °C, and low: 20 °C ≤ x ≤ 30 °C, respectively. At low-temperature levels, the metal ion adsorption increases in the order of Mn^2+^ > Pb^2+^ > Cu^2+^ > Cr^6+^ > Co^2+^ > Zn^2+^ > Ni^2+^ > Cd^2+^ and in the order of Pb^2+^ > Cd^2+^ > Zn^2+^ at the medium level, respectively. Meanwhile, for the high level of temperature, the order was Cd^2+^ > Pb^2+^ > Cr^2+^ > Cu^2+^. Temperature is an important indicator of the exothermic or endothermic nature of the adsorption reaction process [135]. The solution viscosity is reduced with the increase in the solution’s temperature and hence increases the diffusive transport of adsorbate species from the bulk phase to the solid/liquid interface and through pore diffusion [136]. An increase in the adsorption capacity at higher solution temperatures indicates the endothermic nature of the adsorption reaction due an increase in the kinetic transport of adsorbate solutes and a higher diffusional rate [38]. However, a decrease in the adsorption capacity with an increase in the temperature indicates that the reaction has become exothermic, and this is due to the heat-induced decrease in the attractive adsorptive forces between the adsorbate and the adsorbent’s surface [137].

The temperature effect on the agricultural adsorbent’s capacity depends on the surface functional groups [138]. They reviewed and reported the results of many studies, such as mango leaf powder [139], rice husk [139], orange peel [140], and coconut shell [141]), which were all found to increase the percentage adsorption of metal ions with the increase in the temperature range (25–40 °C). In comparison, the adsorption of Cd^2+^ on the cashew nut shell was decreased from 80.13% to 74.32% with the rise in temperature from 30 °C to 60 °C, respectively. Many studies have also reported that metal ion uptake by some adsorbents is reduced with an increasing temperature [133,142].

## 4. Future Perspectives and Future Challenges

To overcome the high costs of commercial activated carbon (CAC) and to overcome the other operational issues that have been associated with the use of CAC as the adsorbent, raw and modified agricultural biomass residue-based adsorbents have gained a significant level of attention as an alternative, carbon-containing, easily accessible, and cost-effective adsorbent in the removal of aqueous phase heavy metal ions with a high degree of binding capacity. From the extensive literature review on adsorption-based wastewater treatment technology, the following points presented are the challenges and future directions that need to be addressed so that adsorption-based technology may be more effective and popularize this technology for the future remediation of water pollution.

Overall economy: the overall economically feasible operation of an adsorption-based treatment plant depends on many factors. Various costs associated with the operating costs, fixed costs, including the installation cost, adsorbent pre-treatment/preparation costs, and cost of adsorbent regeneration are all especially important for determining the feasibility of the full process. Among them, the adsorbent cost alone, including its procession, is above 60% of the total operating cost. Therefore, the adsorbent material selection is crucial for the adsorptive separation process. Various non-conventional solid waste-based adsorbents may be an alternative, cost-effective solution to this process.

Industrial scale problems and lab-based experiments: due to the introduction of various environmental protection laws and regulations, industries have imposed the discharge of waste into the environment. However, industries sometimes discharge harmful chemical waste at a higher than prescribed limit. Therefore, the industry always looks into some low-cost technology like adsorption, and many industries have already adopted this technology. However, the effectiveness of this adsorption-based technology is mainly judged using the laboratory-based batch adsorption results with limited continuous experimental results. Therefore, more continuous adsorption operation results, if possible, along with the pilot-scale results are required before commercial implementation.

Batch and continuous column analysis: based on the literature review over the last two decades, it has been found that more than 80% of adsorption-based studies are of the batch scale. The challenge of adsorption-based studies lies here. These batch studies are confined to various kinetics, isotherm, and thermodynamic analysis with a very small lab scale. The batch-scale study results cannot be adopted directly for industrial use without continuous operation. Several recent studies have come up with some lab or bench-scale continuous studies of adsorption in a packed bed, fluidised bed, and semi-fluidised system to help in the scale up of this process. More research is required in the field of continuous adsorption systems and scale-up processes.

Adsorption modelling: For large-scale operation and process design adsorption modelling, the procedure for the accurate estimation of various kinetic parameters, isotherm models, and the thermodynamic parameters for the multicomponent system are essential.

Adsorbent regeneration and reuse: it has been mentioned previously in that the 60% cost of an adsorption-based system depends on the cost of the adsorbent. Therefore, in the age of sustainable development, adsorbent regeneration must be given significant priority. To reduce waste production, secondary pollution, and operating costs, and to make the overall technology more cost-effective for further reuse, regeneration of the loaded adsorbents is an essential process. Moreover, the capture adsorbate must be recovered as they may be valuable products or to aid in minimising secondary pollution. Hence, an eco-friendly and low-cost alternative regeneration method must be developed to reduce waste production and cost, as well as maximise the cycle number to use for a greater number of times under industrial operations.

Process optimisation: in adsorption-based studies, process optimisation is required under controlled conditions and for further applications in real-field situations. In most cases, the actual process effluents are multicomponent and compete with the adsorbates. The multicomponent systems always reduce the ideal adsorption capacity, meaning therefore that the modelling and optimisation of these multicomponent systems will be quite complex.

## 5. Conclusions

Water pollution due to heavy metal ion contamination resulting from various sources, including untreated industrial effluent discharge and agricultural activities, is of global concern and to find out an efficient but sustainable and cost-effective remediation solution to these important global problems imposes a challenging task on scientists, researchers, and practising engineers. Among the various conventional remediation techniques, adsorption-based separation technology is considered to be one of the most effective approaches widely used in treating heavy metal contaminated water and wastewater due to its simple operation, design simplicity, high separation efficiency, efficiency at lower pollutant concentrations, high selectivity at the molecular level, low energy consumption, ability to separate multiple pollutant components, and minimize secondary pollution. This review article presented a compilation of various scattered literature data along with the up-to-date development batch metal cation adsorption results using a wide range of non-conventional and cost-effective agricultural solid waste-based adsorbents under various process conditions. It is clear from the present literature survey in that non-conventional raw or modified agricultural solid waste-based adsorbents are emerging as effective, but low-cost adsorbents for heavy metal ions present decontamination problems. The utilisation of this large amount of agricultural solid waste-based effective adsorbents in the water and wastewater treatment industries is a sustainable and cost-effective pollution control option alternative to the costly CAC adsorbents. The literature has also revealed that in some cases, the modification of the adsorbent increased the removal efficiency of adsorption. The effective metal removal efficiency from the aqueous phase mainly depends on the adsorbent’s characteristics and various physicochemical process parameters. Therefore, this review article was compiled to critically analyse the large batch adsorption results on heavy metal ion adsorption by the wide ranges of agricultural solid waste-based adsorbents, specifically the adsorbent’s characteristics, and under various influential process parameters, such as the initial adsorbate metal ion concentration, the initial solution pH, the adsorbent doses, and the temperature, respectively.

## Figures and Tables

**Table 1:** Adsorption capacities *q_m_* (mg/g) of several recently reported raw and modified agricultural waste materials for heavy metal ion adsorption.

Agricultural By-Products Raw and Modified/Treated Adsorbents	Adsorbate Heavy Metal Ions	Maximum Monolayer Adsorption Capacity, *q_max_* (mg/g), at Optimum Process Conditions	References
Avocado seed	Cr (VI)	35.5	Ahmet and Danish [50]; Rangel et al. [61]
Jackfruit peels	Cu^2+^ Pb^2+^ Cd^2+^ Mn^2+^	17.5 10.1 20 76.9	Ibrahim et al. [62]; Ayob et al. [63]
Data palm empty fruit bunch	Cr^6+^	70.49	Rambabu et al. [44]
Pineapple peel	Cr^6+^	40	Shakya et al. [64], Yousef et al. [65]
Canola seeds	Pb^2+^ Cd^2+^	44.25 52.36	Affonso et al. [66]; Ayob et al. [63]
*Laurus nobilis* leaves	Cu^2+^ Pb^2+^ Cd^2+^ Zn^2+^	6.04 96.15 8.6 8.74	Gumus et al. [43]; Ogunlalu et al. [31]
*Vigna radiata* husk biomass	Cu^2+^ Co^2+^ Ni^2+^	11.05 15.04 19.88	Naseem et al. [67]
Coffee pulp	Cr^6+^	13.48	Ayob et al. [63]
*Cajanus cajan* Husk	Cd^2+^	42.16	Devani et al. [68]; Sazali et al. [69]
Orange peel	Cd^2+^	170.3	Chen et al. [70]
Litchi peel	Cd^2+^	230.5	Chen et al. [70]
Date seed biochar	Ni^2+^	19.54	Mahdi et al. [71]
Avocado peel	Pb (II) Ni (II)	4.93 9.82	Ahmet and Danish [50]; Mallampati, [72]
Modified peanut shell	Hg(II)	30.72	Sulyman et al. [60]
Coconut husk	Cu^2+^ Ni^2+^ Pb^2+^ Zn^2+^	443.0 404.5 362.2 338.0	Malik and Dahiya [73]
Orange peel	Pb (II)	204	Sulyman et al. [60]
Banana peels	Cu^2+^ Ni^2+^ Pb^2+^	14.3 27.4 34.5	Thuan et al. [74]; Ayob et al. [63]
Corn straw	Cd^2+^ Pb^2+^	38.91 28.99	Chi et al. [75], Yousef et al. [65], Yan et al. [76]
Pomegranate peel	Cu^2+^	30.12	Ben-Ali et al. [55]
Modified activated bamboo	Cd^2+^	202.55	Zhang et al. [77]; Sazali et al. [69]
Orange peel	Cu^2+^	63.3	Guiza [78]
Flax fiber tows	Cu^2+^ Pb^2+^ Zn^2+^	9.92 10.74 8.4	Abbar et al. [79]
Eucalyptus bark	Zn (II)	131.6	Afroze et al. [49]
Banana peel	Cd^2+^ Pb^2+^	5.71 2.18	Gisi et al. [5]
Sweet potato peel	Pb^2+^	18	Asuquo et al. [80]
Peanut husk	Ni^2+^	56.82	Abdelfattah et al. [81]
Orange peel	Hg^2+^	7.46	Chinyelu [82]
Tomato leaf	Ni (II)	58.8	Gutha et al. [83]
Rapeseed waste	Zn (II)	13.9	Paduraru et al. [84]
Jackfruit leaf	Ni (II)	11.5	Boruah et al. [85]
Sorghum hulls	Cu^2+^	148.93	Imaga, Abia et al. [86]
Coffee residues	Pb^2+^, Zn^2+^	9.7 (Pb^2+^), 4.4 (Zn^2+^)	Wu, Kuo et al. [28], Utomo and Hunter [87]
Modified Okra biomass	Cu^2+^, Zn^2+^, Cd^2+^, Pb^2+^	72.72 (Cu^2+^), 57.11 (Zn^2+^), 121.51 (Cd^2+^), 273.97 (Pb^2+^)	Singha and Guleria [88]
Sugarcane bagasse	Mn^2+^	0.423	Anastopoulos et al. [51]
Sugarcane bagasse	Cd^2+^	0.955	Moubarik and Grimi [89], Anastopoulos et al. [51]
Peanut shell	Pb^2+^	39	Tasar et al. [47]
Pistachio hull waste	Hg^2+^	48.78	Rajamohan [90]
Coconut tree sawdust	Cu (II) Pb (II) Zn (II)	3.9 25.0 23.8	Putra et al. [91]
Modified rice husk	Hg^2+^	89	Song et al. [92], Yousef et al. [65]
Modified Sugarcane bagasse	Cu^2+^	30.9	Rana et al. [17]
*Garcinia cambogia* plants	As	704.11	Gautam et al. [93]
*Oryza sativa* plants	Cd^2+^	20.70	Gautam et al. [93]
Corn stover	Cr^2+^	84	Gautam et al. [93]
Palm tree branches	Cr^+4^	157	Guat et al. [2]
Egyptian mandarin peel (raw)	Hg^2+^	19.01	Husein et al. [94]; Gisi et al. [5]
Raw sugarcane bagasse	Hg^2+^	35.71	Khovamzadeh et al. [95]; Anastopoulos et al. [51]
Orange peel	Cu^2+^, Pb^2+^, Zn^2+^	70.73 (Cu^2+^), 209.8 (Pb^2+^) and 56.18 (Zn^2+^)	Feng and Guo [16] Gomez-Al [96]
Barley straw (raw)	Cu^2+^	4.64	Gisi et al. [5]
Garden grass (raw)	Pb^2+^	58.34	Gisi et al. [5]

**Table 2:** The effects of several agricultural solid waste-based adsorbent characteristics on heavy metal ion adsorption.

Adsorbents	Contaminants (Heavy Metals and Dyes)	Characterisation Properties					References
		Specific Surface Area/BET(m^2^/g)	Particle Size Distribution	Elemental Analysis (%)	FTIR Analysis	pH_pzc_	
Pinecone	Cd^2+^, Cu^2+^, Pb^2+^	0.2536	50 µm	-	O-H, C-H, -CH_2_, C=O	6.2	Dawood et al. [97] Marawa et al. [98]
Avocado seed	Cr^6+^	1.75	0.1–1.5 mm		O-H group -CH_2_ stretching	6.4	Bazzo et al. [99]; Leite et al. [100]
HAS avocado shell?	Ni^2+^		-	43.13 (carbon), 7.17 (hydrogen), 48.35 (oxygen), 0.66 (nitrogen) and 0.89 (sulphur)	C==O, O-H, -CH_2_ stretching	6.8	Garcia and Cristiani-Urbina, [101]
Raw pomegranate peel	Cu^2+^	598.78	205 µm, 850 µm and 2375 µm		C=O in carboxylic acid, acetate groups -COO, ketone, C–O groups of carboxylic acid, alcoholic, phenolic, ether and ester groups.		Ben-Ali et al. [55]
Sugarcane bagasse pith (sulphurised activated carbon)	Zn^2+^	500	-	9.10 (sulphur) and 5.20 (ash)	S==O, and C-S vibrations	4.3	Krishnan et al. [102]
Jack fruit leaf powder	Ni^2+^	246.9	-	-	-OH groups, -CH_2_ group, and CO bonds and C=S bonds.	-	Boruah et al. [85]
Coffee residues	Pb^2+^, Zn^2+^	0.19	-	-	-	3.9	Wu et al. [28]
Guava leaves (activated)	Cd^2+^	100.76	Pore volume 0.415 cm^3^/g and pore diameter 47.091 Å	-	O–H, C–H, C=C and –SO_3_ bonds	-	Abdelwahab, Fouad et al. [81]
DateStones Pd^2+^Cd^2+^		950 950					Sulyman et al. [60]
Olive stone Hg^2+^		400–850	-				Wahby et al. [103]

**Table 3:** The selected reported list on the effect of changes in the adsorbent dosages on the percentage of adsorptive metal ion removal using several agricultural wastes as adsorbents during the last 10-year period.

Adsorbents	Adsorbates (Heavy Metals)	Adsorbent Dosage	Trend on Percentage (%) Removal Range	References
*Brassica campestris* agricultural waste	Ni^2+^, Pb^2+^ Cr^6+^ 0.2–1 g/L		Increase	Shaikh et al. [116]
Mango kernel (bio-composite)	Cr (VI)	0.05–0.3 g/L	Decrease	Akram et al. [117]
Bagasse (activated)	Cr	0.5–1.5 g/L	Increase	Olayebi et al. [118]
Croncob (activate)	Cr	0.5–2.4 g/L	Increase	Olayebi et al. [118]
Bagasse (activated)	Fe^3+^	0.5–2.5 g/L	Increase	Olayebi et al. [118]
Croncob (activated)	Fe^3+^		Increase	Olayebi et al. [118]
Banana peel biochar	Pb^2+^	0.5–3.0 g/L 0.01–0.2 g/L	Increase	Zhou et al. [119]
Eucalyptus *sheathiana* bark	Zn^2+^	0.01–0.03 g	Decrease	Afroze et al. [49]
Bagasse pith (sulphurised activated carbon)	Zn^2+^	0.5–8 g L^−1^	Increase	Krishnan et al. [102]
Jackfruit leaf powder	Ni^2+^	1–5 g L^−1^	Decrease	Boruah et al. [85]
Sugarcane bagasse (sulphuric acid-treated)	Cu^2+^	0.5–2 gm/100 mL	Increase	Rana et al. [17]
Grapefruit peel	Cd^2+^, Ni^2+^	1–4 g L^−1^	Increase	Torab-mostaedi et al. [120]
Tamarind fruit shell	Ni^2+^	0.01–0.08 g/10 mL	20–90	Pandharipande and kalnaka [121]
Almond shell biocar	Ni^2+^ Cd^2+^	0.1–10 g/L 0.1–10 g/L	Increase	Kilic et al. [112
Rice husk	Pb^2+^, Cd^2+^ Cu^2+^, Ni^2+^	0.02–0.06 g/L	Increase Increase	Hegazi [122]

## Data Availability

Data sharing not applicable to this article as no datasets were generated or analysed during the current study.
